# Investigation of lateral geniculate nucleus volume and diffusion tensor imaging in patients with normal tension glaucoma using 7 tesla magnetic resonance imaging

**DOI:** 10.1371/journal.pone.0198830

**Published:** 2018-06-07

**Authors:** Manuel A. Schmidt, Michael Knott, Robin Heidemann, Georg Michelson, Tobias Kober, Arnd Dörfler, Tobias Engelhorn

**Affiliations:** 1 Department of Neuroradiology, University Hospital Erlangen-Nürnberg, Schwabachanlage 6, Erlangen, Germany; 2 Siemens Healthcare GmbH, Diagnostic Imaging, MR Technology & Research Systems, Allee am Röthelheimpark 2, Erlangen, Germany; 3 Department of Ophthalmology, University Hospital Erlangen-Nürnberg, Schwabachanlage 6, Erlangen, Germany; 4 Advanced Clinical Imaging Technology (HC CMEA SUI DI BM PI), Siemens Healthcare AG, Lausanne, Switzerland; Bascom Palmer Eye Institute, UNITED STATES

## Abstract

**Background:**

There is evidence that glaucoma is a neurodegenerative disease involving the whole visual pathway. We prospectively examined potential benefits of volumetry of the lateral geniculate nucleus (LGN) and diffusion tensor imaging (DTI) using a new 7T scanner.

**Methods:**

20 patients with normal tension glaucoma and 16 control individuals were examined. LGN volume and fractional anisotropy (FA) of the optic tract (OT) and the optic radiation (OR) and their correlation with RNFL (retinal nerve fiber layer) thickness were analyzed.

**Results:**

LGN volume was significantly reduced in NTG (60.9 vs 88.3; p < 0.05). FA of the OT (right: 0.35 vs 0.66, left: 0.36 vs 0.67; p < 0.05) and of the OR (right: 0.41 vs 0.70, left: 0.41 vs 0.69; p < 0.05) was also significantly reduced. Nasal RNFL thickness correlated with the volume of the contralateral LGN (r = 0.471, p = 0.05). Temporal RNFL thickness correlated with the volume of the ipsilateral LGN (r = 0.603, p = 0.015).

**Conclusion:**

NTG leads to significant atrophy of the LGN compared to controls. FA of the optic tract and the optic radiation is reduced in NTG as sign of axonal degeneration. RNFL thickness but not FA correlates with LGN volume.

## Introduction

Magnetic resonance imaging has been used to analyze the visual pathway in glaucoma; including morphological examinations [1, 2] as well as functional measurements by means of diffusion tensor imaging (DTI) [3, 4]. Thereby, glaucoma is more and more classified as a neurodegenerative disease [5] and previous histopathological examinations demonstrated degenerative tissue changes of the intracranial optic nerve, the lateral geniculate nucleus (LGN) as well as the optic cortex [6] and even in structures outside the visual pathway such as the amygdala [7] in primary open-angle glaucoma. These degenerative processes can also be found in vivo by MRI/DTI as atrophy of the LGN [8, 9] as well as reduced fractional anisotropy (FA) of the optic nerve [10, 11] have been found. Regarding volumetry of the LGN, even ultra high-field (UHF) MRI data have been analyzed and revealed atrophy of the LGN in primary open-angle glaucoma (POAG) [12, 13]. The potential benefit of a higher field strength is a higher signal to noise ratio (SNR), which can be used to increase the spatial resolution [14] resulting in a higher accuracy in delineating such small structures as the LGN. Examinations of LGN atrophy in glaucoma were focused on POAG as the most common etiology in the past. The idea of glaucoma being always related to an elevated intraocular pressure (IOP) with the mechanistic explanation of pressure induced damage of the optic nerve is widespread, however, up to 40% of glaucoma patients in Caucasian populations present with an IOP within the physiological range (≤ 21 mmHg). This subtype of normal tension glaucoma (NTG) shows the same peripapillary retinal nerve fiber layer (RNFL) thinning and subsequent visual field defects as the high pressure glaucoma phenotype [15].

The pathophysiological essentials of NTG are not well understood. Neurodegeneration beyond the visual pathway has been shown in NTG [16], but further investigation, e.g. correlation with ophthalmological measures of disease severity, is necessary. This is especially important because the most noticeable feature of glaucomatous optic neuropathy, the elevated IOP is absent in NTG, making the disease difficult to diagnose.

Aim of our study was to assess the integrity of the optic tract and the optic radiation by selective tractography based on high resolution DTI and to evaluate LGN atrophy in NTG using 7T UHF-MRI to investigate the potential to complement ophthalmological diagnosis of NTG.

## Material and methods

### Subjects

We included 20 patients with a history of binocular NTG (mean age +/- SD = 55y +/- 8.7) who were recruited through the Department of Ophthalmology of the University Hospital Erlangen-Nuremberg. All patients underwent full ophthalmological examination including perimetry and measurement of RNFL.

Intraocular pressure was < 21 mmHg in all patients. Patients with severe neurological conditions such as (minor and major) stroke or postischemic defects, inflammatory CNS diseases and intracranial masses were excluded. The control group comprised 16 healthy volunteers (mean age +/- SD = 47y +/- 9.5) without any signs of glaucoma. The Clinical Investigation Ethics Committee of the University of Erlangen-Nuremberg approved the protocol of this prospective study and the research was conducted in accordance with the Declaration of Helsinki. All participants gave written informed consent prior to all measurements.

### RNFL measurements

Current RNFL data (not older than 6 months regarding the date of image acquisition) was available for 13 of the 20 NTG patients.

We used spectral domain optical coherence tomography (SD-OCT; Spectralis OCT, Heidelberg Engineering GmbH, Heidelberg, Germany). For SD-OCT, simultaneous measurements of multiple wavelengths of reflected light across a spectrum are used to produce detailed, cross-sectional 3D images of the retina. An automatic positioning system creates an anatomical map of every patient based on the position of the fovea and the center of optic nerve head. The neuroretinal rim area is measured in 6 sectors (temporal superior, nasal superior, nasal, nasal inferior, temporal inferior and temporal) as well as globally. RNFL thickness is measured as the difference between the inner margin of the ILM (internal limiting membrane) and the outer margin of the RNFL and the obtained values are automatically compared to a normative database [17, 18].

### Imaging protocol

We used a 7T ultra high-field scanner (Magnetom Terra, Siemens Healthineers, Erlangen, Germany) with a gradient field strength of up to 80 mT/m with a slewrate of 200 T/m/s. For signal reception, a 32-channel receive array head coil (Nova Medical, Wilmington, MA, USA) was used.

**Fig 1:**
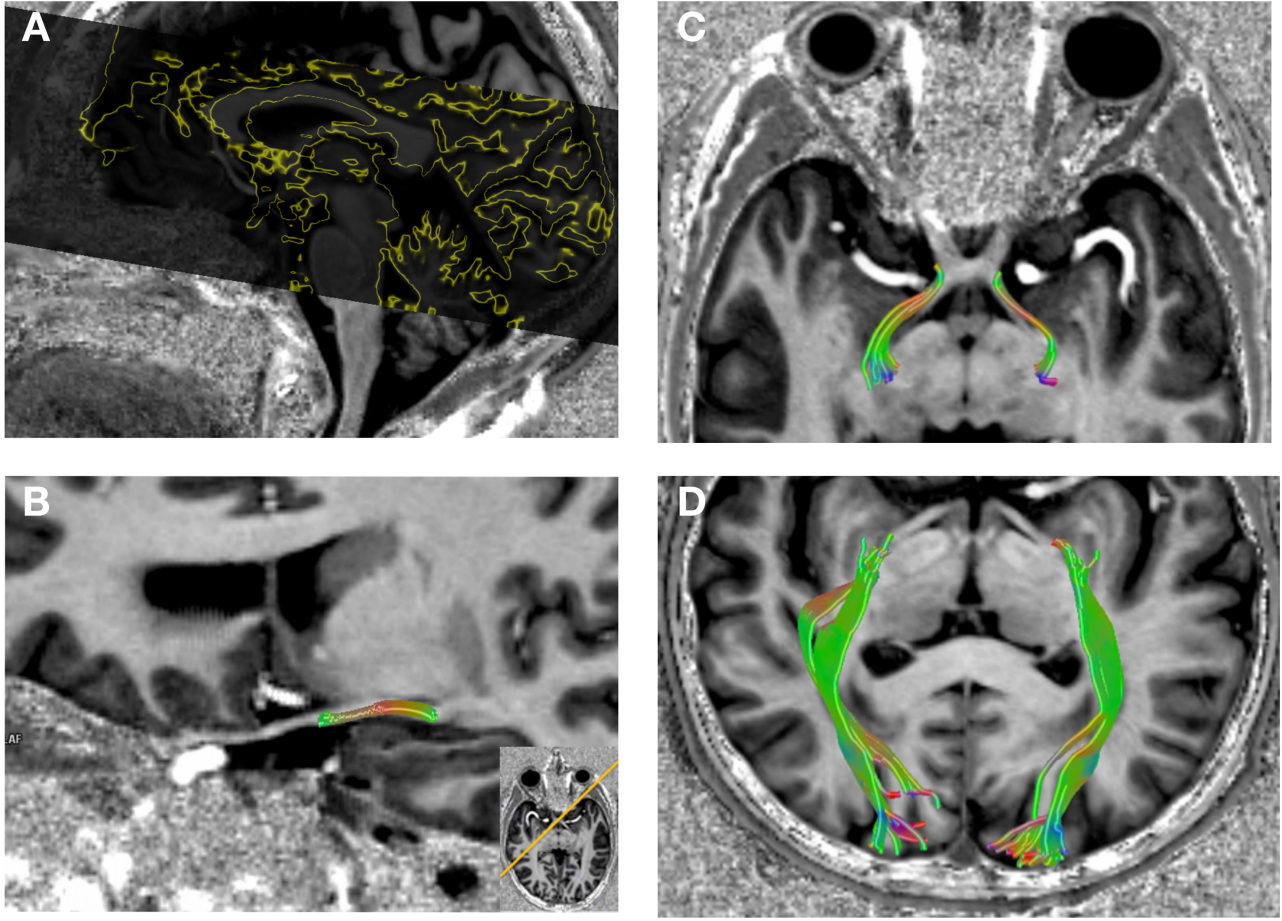
DTI coverage of the visual pathway (A). Reconstruction of the DTI dataset as selective tractography of the optic tract (B and C) and the optic radiation (D) in NTG.

Axial high resolution DTI was performed with 1.3 mm isotropic resolution using a readout-segmented echo planar imaging sequence [19] (TR = 7700 ms, TE = 57 ms, echo-spacing = 0.36 ms, 5 segments, FoV = 220 x 220 mm^2^) resulting in a slab of 66.3 mm with 51 contiguous slices covering the whole visual pathway (Fig 1). Diffusion weighting was carried out with a maximal b-value of 1000 s/mm^2^ along 20 directions complemented by one scan with a b-value of zero. Manually adjusted B0-shimming was optimized to reduce distortion artifacts, especially regarding the suprasellar cistern.

Anatomical information was obtained through a high-resolution 3D MP2RAGE (Magnetization Prepared 2 Rapid Acquisition Gradient Echoes) [20] which improves the gray/white matter contrast (0.8 mm isotropic resolution, TR = 4530 ms, TE = 1.92 ms, TI1 = 700 ms, TI2 = 2700 ms, FoV = 226 x 240 mm^2^).

For comparison, all patients received a 3D MPRAGE (Magnetization Prepared Rapid Acquisition Gradient Echo) with a clinical 3T scanner (Magnetom Trio, Siemens Healthineers, Erlangen, Germany). Sequence parameters were as follows: slice thickness = 0.8 mm, TR = 2400 ms, TE = 3.59 ms, TI = 900 ms, FoV = 256 x 256 mm^2^.

### Image analysis and statistics

LGN was identified on coronal slices on the MP2RAGE in thin slice data above the hippocampus and the temporal horn of the lateral ventricle, beneath and lateral to the thalamus, and medial to the optic radiation as previously described [12, 21, 22]. LGN volumes were measured manually by circling its cross-sectional area by hand on every slice it was visible (3 to 5 slices) by using a commercially available software plug-in (Neuro 3D, syngo.via, Siemens Healthineers, Erlangen, Germany). The measured areas of the LGN in each slice were then summed up and multiplied with the slice thickness to derive the volume in mm^3^. Measurements were obtained by a neuroradiologist and performed twice, average values of both measurements were used for statistical analysis.

Selective deterministic tractography followed by region-of-interest analysis was performed to obtain FA values for the optic tract and the optic radiation. We chose to examine the optic tracts and not the optic nerves to avoid susceptibility artifacts of the anterior clinoid processes impairing the tractography results. For that, the DTI dataset was fused with the high resolution MP2RAGE for anatomical information and manual seed regions of interest (ROIs) were set as following: 1. for the optic tract immediate behind the optic chiasm to the LGN for each side separately 2. for the optic radiation from the LGN to the optic cortex for both sides, respectively (Fig 1).

Normal distribution of the variables was assessed by a Kolmogorov-Smirnov test.

Unpaired Student’s t-tests were performed to describe differences in LGN volume, FA of the optic tract and optic radiation as well as RNFL thickness.

Pearson product-moment correlation coefficient was used for correlation analysis between LGN volumes, FA of the optic tract and optic radiation and RNFL thickness in NTG (without correction for multiple comparisons). P values ≤ 0.05 were considered statistically significant. All statistical analyses were performed using SPSS version 19.0 (SPSS, Inc., Chicago, IL, USA).

## Results

RNFL (retinal nerve fiber layer) thickness was significantly reduced bilaterally in 12 patients compared to an age-matched normative database provided through the device software by the manufacturer (Spectarils OCT, see above). In one patient reduced RNFL was limited to the right eye. RNFL measurements are summarized in Table 1.

**Table 1:** RNFL thickness and LGN volumes of NTG patients. Significant correlation of the nasal sector RNFL thickness (crossing axons of the ganglion cells in the optic chiasm) with the volume of the contralateral LGN. In contrast, the temporal sector RNFL thickness (non-crossing axons of the ganglion cells in the optic chiasm) correlates with the volume of the ipsilateral LGN.

**RNFL thickness, μm, right eye**	**LGN volume / mm^3^**	ST*	T*	**IT**	**IN**	**N**	SN*
patient no							
1	70.4	95	34	50	86	55	107
2	80	119	74	145	53	27	66
3	46.8	81	58	106	69	66	50
4	60	59	127	62	11	38	74
5	79.2	68	51	31	55	59	92
6	75.2	75	36	47	32	39	62
7	62.4	99	54	97	83	49	95
8	39.2	115	74	87	50	26	55
9	39	98	51	80	69	57	88
10	50.4	61	35	99	69	62	57
11	47.2	37	30	29	56	42	46
12	64	80	88	70	52	54	60
13	48.8	56	30	91	98	64	72
**RNFL thickness, μm, left eye**		ST*	T*	**IT**	**IN**	**N**	SN*
patient no							
1	83.2	104	64	128	96	56	105
2	88.8	125	66	80	117	50	79
3	85.6	88	62	138	84	66	27
4	60	46	49	63	67	51	52
5	84.8	90	71	68	54	56	112
6	65.6	85	37	43	48	49	90
7	36.8	55	42	107	55	38	43
8	50.4	122	76	107	62	34	79
9	46.4	84	44	82	62	54	75
10	61.6	107	51	111	64	62	80
11	60.8	62	42	54	69	34	51
12	60	82	39	47	46	35	49
13	43.2	54	27	58	81	69	57

RNFL = retinal nerve fiber layer, ST = superior temporal, T = temporal, IT = inferior temporal, IN = inferior nasal, N = nasal, SN = superior nasal.

^*^ indicates significant correlations (p < 0.05).

**Fig 2:**
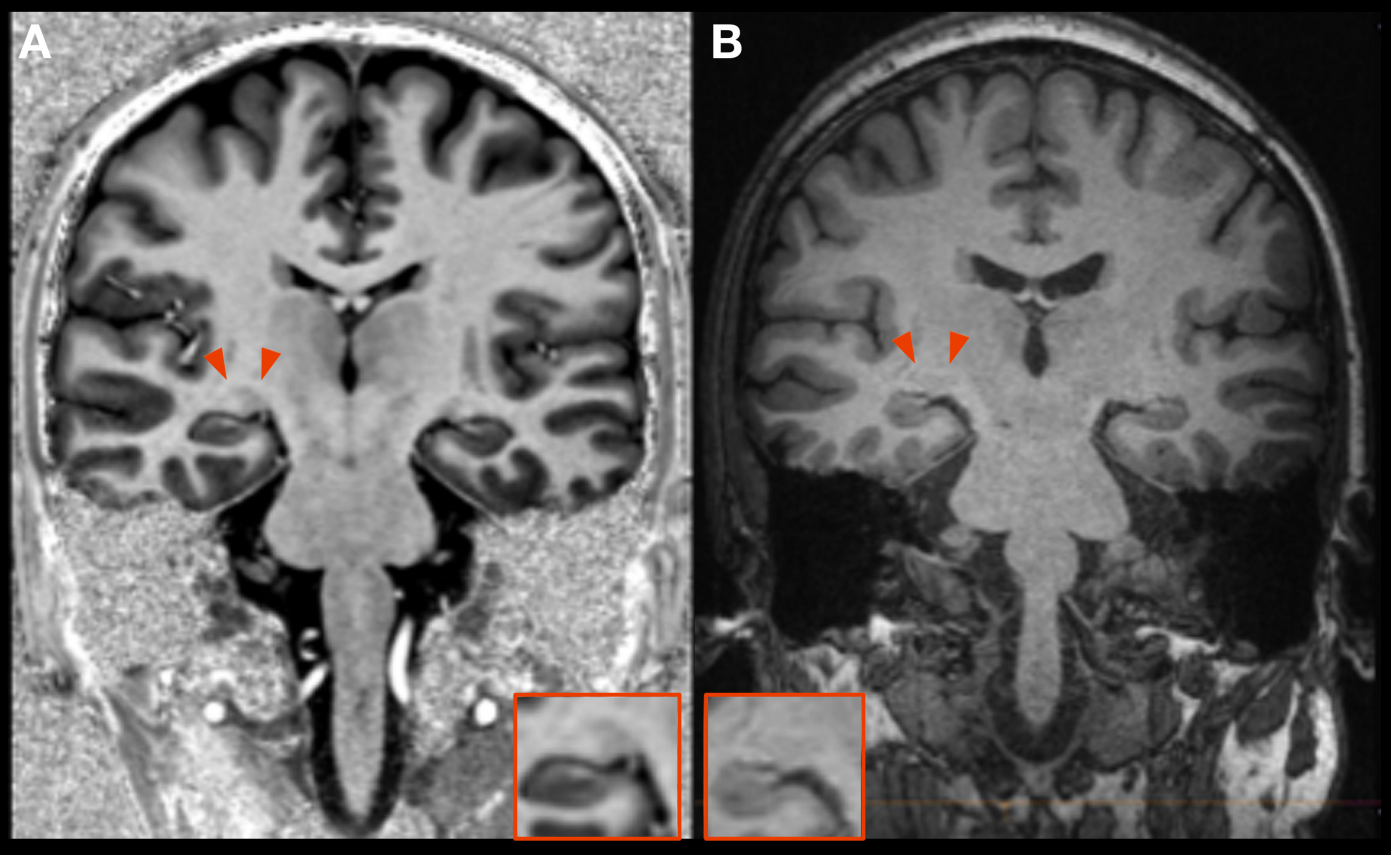
Comparison of LGN imaging at 7T and 3T. The LGN can be clearly identified on a coronal MP2RAGE (A) at 7T whereas this is not the case on a standard MPRAGE at 3T (B).

**Fig 3:**
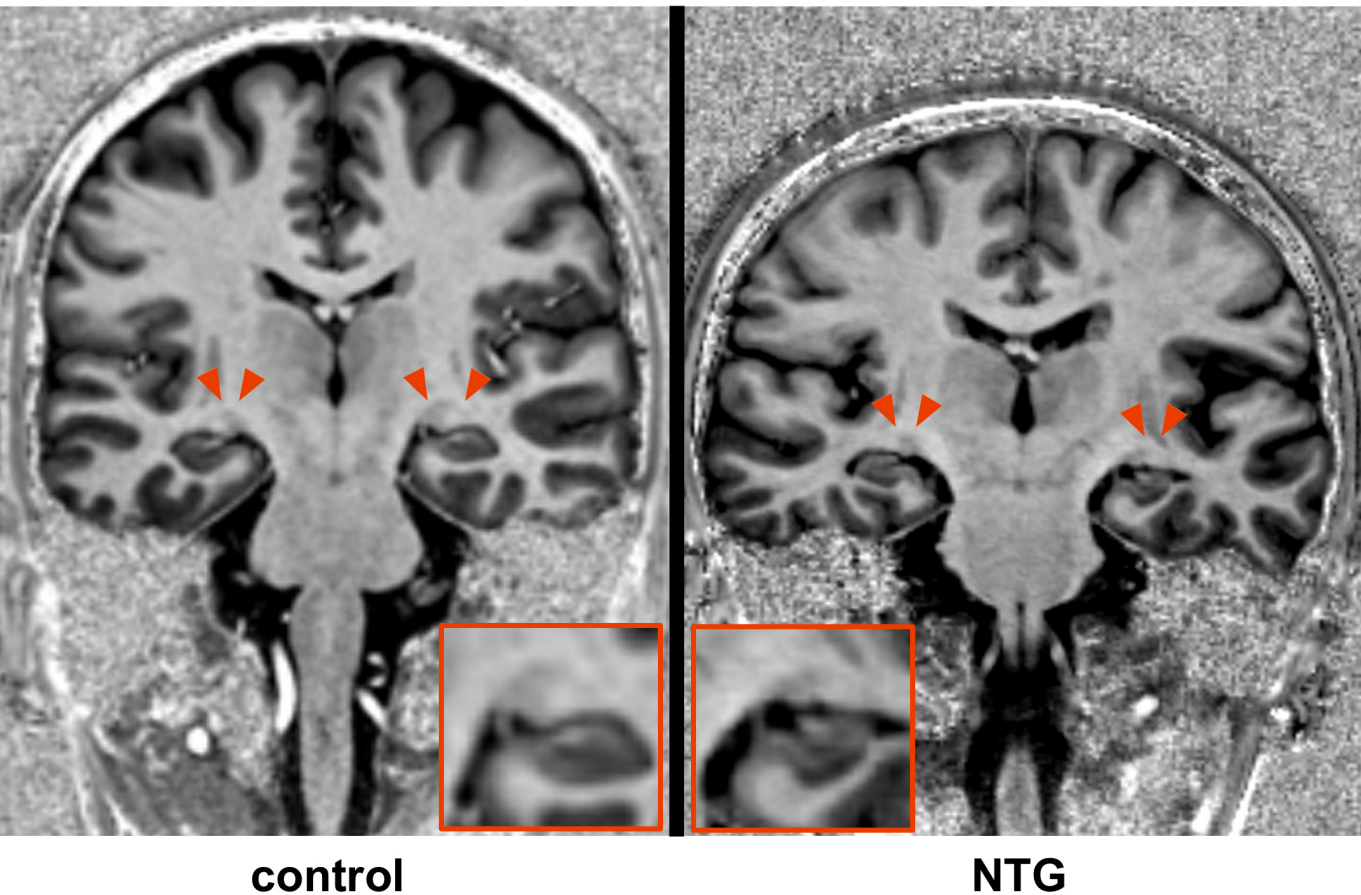
Distinct atrophy of the LGN (red arrows) in NTG compared to healthy control.

**Fig 4:**
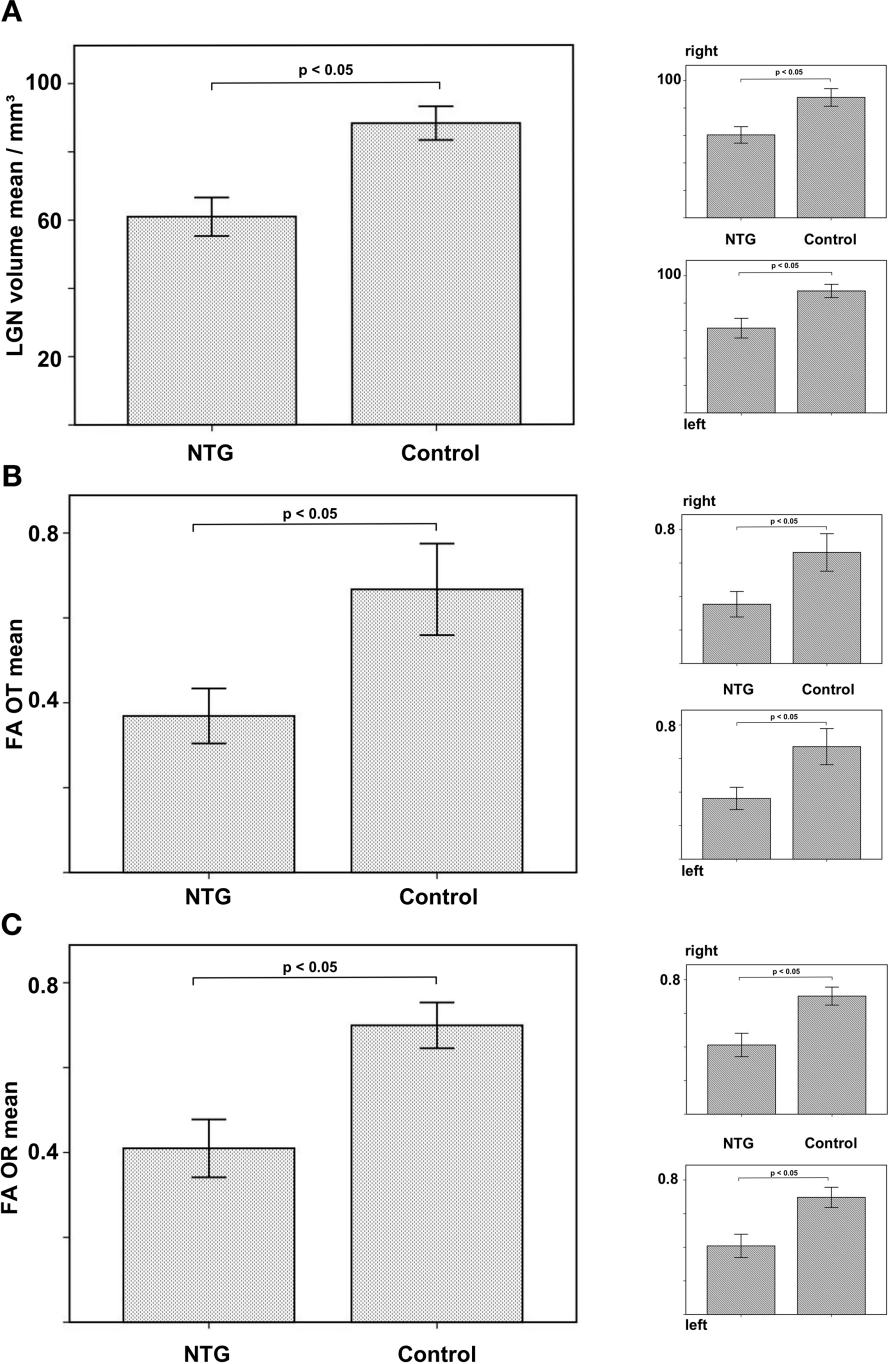
LGN volume in NTG compared to controls (A). FA of the optic tract (OT) in NTG compared to controls (B). FA of the optic radiation (OR) in NTG compared to controls (C).

The LGN can be superiorly delineated at a field strength of 7T compared to standard imaging at 3T (Fig 2). LGN volume was significantly reduced in NTG on the right (60.4 mm^3^ vs 87.7 mm^3^, p < 0.05) and reduced on the left (61.8 mm^3^ vs 88.8 mm^3^, p < 0.05; combined right and left 60.9 vs 88.3, p = 0.05; Figs 3 and 4A). There was a strong positive correlation of LGN volumes of both sides in NTG (r = 0.768, p = 0.001).

Regarding selective tractography, FA of the optic tract was significantly reduced compared to controls (right 0.35 vs 0.66, p < 0.05; left 0.36 vs 0.67, p < 0.05; combined 0.37 vs 0.67, p < 0.05; Fig 4B). FA of the optic radiation was also significantly reduced in NTG (right 0.41 vs 0.70, p < 0.05; left 0.41 vs 0.69, p < 0.05; combined 0.41 vs 0.69, p < 0.05; Fig 4C).

Correlation analysis revealed a significant positive correlation of LGN volume and RNFL thickness. The nasal sector RNFL thickness (superior nasal sector, SN) correlated with the volume of the contralateral LGN (r = 0.471, p = 0.05), whereas the temporal sector RNFL thickness (superior temporal, ST and temporal, T) correlated with the volume of the ipsilateral LGN (r = 0.509, p = 0.038 and r = 0.603, p = 0.015).

**Fig 5:**
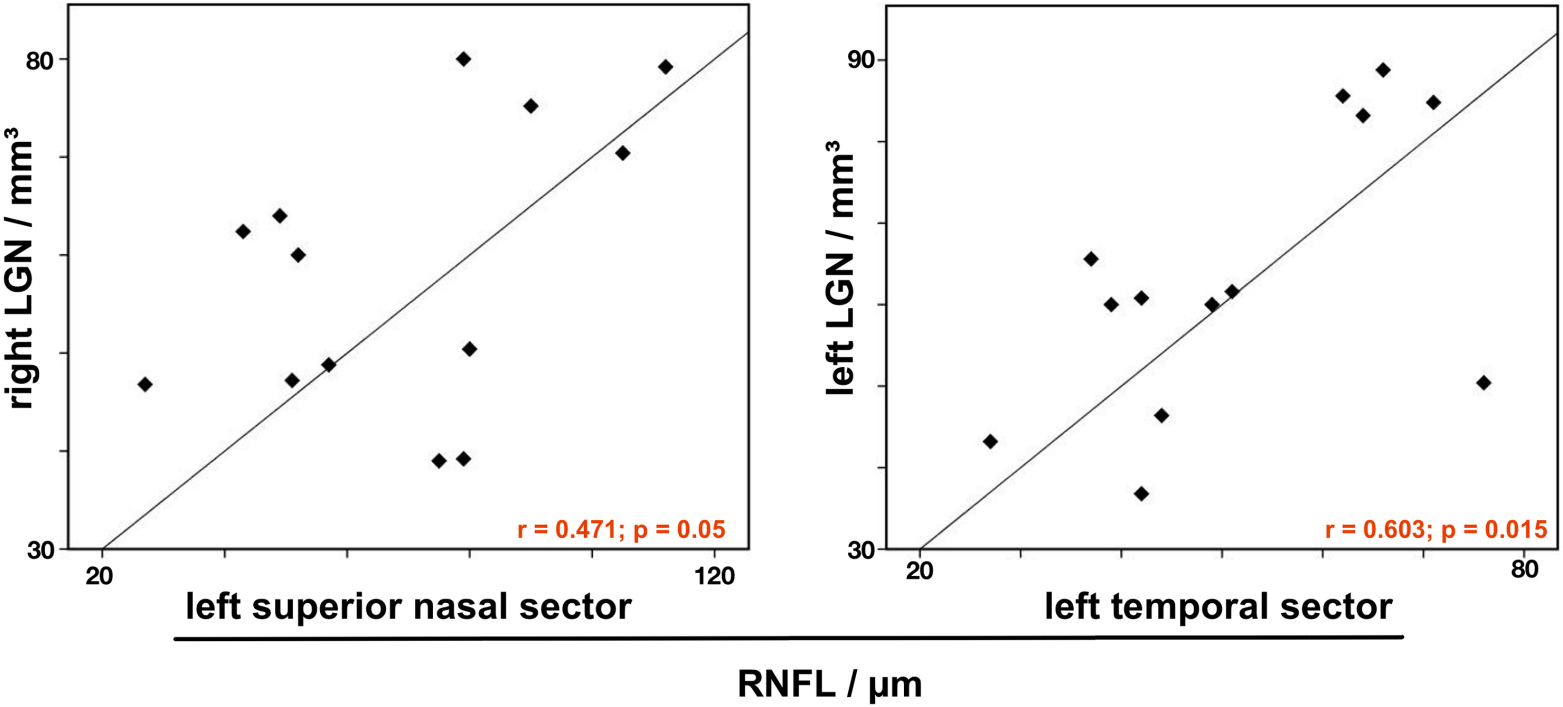
Correlation analysis reveals a strong correlation of RNFL thickness and LGN volume in NTG according to the complex anatomy of the optic tracts and the optic chiasm. The nasal sector RNFL thickness (crossing axons of the ganglion cells in the optic chiasm) correlates with the volume of the contralateral LGN, whereas the temporal sector RNFL thickness (non-crossing axons of the ganglion cells in the optic chiasm) correlates with the volume of the ipsilateral LGN.

Fig 5 shows scatter plots of the correlation between LGN volume and RNFL thickness. There was no correlation between LGN volume and FA of the optic tract or the optic radiation and no correlation of RNFL thickness and FA values of the visual pathway.

## Discussion

To the best of our knowledge, these are the first ultra high-field MRI data of NTG patients obtained at a magnetic field strength of 7T.

Our results reveal significant atrophy of the LGN in NTG compared to healthy controls. Furthermore, we demonstrate reduced FA of the optic tract in the NTG group as a marker of axonal degeneration.

The LGN, located in the metathalamus, is the relay station of the intracranial visual pathway and of utmost importance providing the interconnection of the 3^rd^ (optic nerve and optic tract) and the 4^th^ neuron (optic radiation). In an experimental primate model of glaucoma, atrophy of the LGN has been described firstly: Yücsel et. al reported degenerative changes in magnocellular, parvocellular and koniocellular pathways in the LGN and these changes were related to intraocular pressure and the severity of optic nerve damage [23].

The high spatial resolution at 7T imaging allows for accurate and possibly superior delineation of the LGN based on high-resolution anatomical datasets compared to standard imaging at 3T with the same acquisition time (Fig 2). Thus, direct volumetry of the LGN was possible without the need for further post-processing. Our results show distinct atrophy of the LGN (Figs 3 and 4A) and are in accordance with the single study that investigated LGN atrophy in NTG so far [24]. However, this previous study has certain limitations. MRI data was obtained at a magnetic field strength of 1.5T and the volume of the LGN could not be directly estimated, requiring extensive postprocessing and the use of atlas information for identification and volumetry of the LGN. Instead, we used a superior MP2RAGE sequence which has an improved gray/white matter contrast allowing to identify the LGN [25]. Together with improved signal-to-noise ratio and higher spatial resolution due to the higher magnetic field strength of 7T, LGN volumes could be obtained during clinical reading of the examination without extensive post-processing. The merit of the higher field strength is a higher signal to noise ratio, which allows for accurate delineation of the LGN without increasing the acquisition time of the anatomical dataset.

We analyzed the correlation of LGN atrophy with the glaucoma stage described by RNFL thickness, which is a reliable measurement and is widely used to classify glaucoma stage [26]. RNFL was significantly reduced in NTG providing an objective measurement of disease severity in these patients.

There was a strong positive correlation of LGN volume and RNFL thickness (Fig 5). The anatomy of the optic tracts and the optic chiasm is complex. In humans, the axons of the ganglion cells of the temporal half of the retina do not cross in the optic chiasm and transmit their signal to the ipsilateral LGN. In contrast, the axons of the ganglion cells of the nasal half of the retina decussate in the optic chiasm and transmit their signal to the contralateral LGN. Accordingly, the temporal RNFL thickness (superior temporal and temporal retinal sectors) correlated with the volume of the ipsilateral LGN, whereas the nasal sector RNFL thickness (superior nasal sector) correlated with the volume of the contralateral LGN.

Our data shows, that degeneration patterns of the LGN in normal tension glaucoma correspond to retinal degenerative processes regarding the anatomy of the visual pathway. Thus, high-resolution imaging of the LGN may complement glaucoma diagnosis and follow-up and may give new insights into the pathophysiology of these degenerative processes when combined with functional imaging. Especially UHF-MRI of the visual pathway is promising, because no further post-processing for volumetry of the LGN is required due to the high spatial resolution. Today, UHF scanners (7T and above) are limited to few specialized research centers. The first clinical 7T scanners are expected to receive FDA approval in the near future, and as soon as they are established, UHF-MRI will become more widely available. All patients suffered from binocular NTG and except for one patient RNFL was significantly reduced in both eyes (Table 1). Degeneration of the LGN and its correlation with disease severity has also been found in primary open-angle glaucoma [8, 9, 12].

Given degenerative volume loss of the RNFL and the LGN, we performed selective tractography of the optic tract and the optic radiation to assess axonal damage. The high spatial information of the acquired MP2RAGE anatomical sequence allows the identification of the LGN, resulting in more precise diffusion measurements [27]. To further improve ROI based diffusion measurements, we performed selective tractography of the optic tracts and the optic radiations. Fractional anisotropy of the axons of the 3^rd^ neuron, i.e. the optic tract, was significantly reduced compared to healthy controls (Fig 4B). Our results are in agreement with earlier measurements of FA of the optic nerves in NTG [24]. Reduced FA of the optic tracts implies axonal damage of the 3^rd^ neuron and is linked to HRT-based indices of glaucoma severity and impairment of the spatial-temporal contrast sensitivity [28].

Regarding the axons of the 4^th^ neuron, there was significantly reduced FA of the optic radiations in patients compared to controls. Degeneration of the 4^th^ neuron has been described before [16]. We assume a—potentially time dependent—anterior-posterior gradient of axonal degeneration of the visual pathway in NTG. The concept of transsynaptic degeneration in glaucoma has been described by Gupta et al. [5]. This concept may apply for NTG patients with progressed disease leading to degeneration of the optic radiation in late stages.

This study has limitations. Although data was obtained with an ultra high-field scanner delivering very high anatomical resolution, further studies comprising more patients need to be performed for characterization of degeneration of the optic radiation in late stages of the disease. One drawback is that control subjects were not age-matched. Therefore, we cannot exclude effects of ageing in our results. However, regarding comparison to healthy controls, our results corroborate previously reported data. Additionally, LGN atrophy could only be correlated with glaucoma stage determined by RNFL thickness rather than histopathological morphology. Therefore, human postmortem studies would be beneficial.

## Conclusion

UHF-MRI including DTI of the visual pathway reliably reveals axonal degeneration of the optic tract and optic radiation in NTG as well as atrophy of the LGN correlating with disease severity measured by RNFL thickness loss. UHF-MRI can thus complement ophthalmological diagnosis of NTG and potentially serve as a powerful tool for treatment monitoring and follow-up.
